# A Twelve-Hundred-Year Stable Oxygen Isotope Chronology Constructed Using Subfossil Wood from Schwarzensee Lake, Austrian Alps

**DOI:** 10.1017/RDC.2022.91

**Published:** 2022-12-20

**Authors:** Marzena Kłusek, Michael Grabner, Jacek Pawlyta, Sławomira Pawełczyk

**Affiliations:** 1 Graduate School “Human Development in Landscapes”, Christian-Albrechts-Universität zu Kiel (CAU), Leibnizstraße 3, 24118 Kiel, Germany; 2 Institute of Physics – Centre for Science and Education, Silesian University of Technology (SUT), ul. Konarskiego 22B, 44-100 Gliwice, Poland; 3 Institute of Wood Technology and Renewable Materials, University of Natural Resources and Life Sciences, Vienna (BOKU), Konrad Lorenz-Straße 24, 3430 Tulln an der Donau, Austria; 4 Faculty of Geology, Geophysics, and Environmental Protection, University of Science and Technology (AGH), 30 Mickiewicza Avenue, 30-059 Krakow, Poland

**Keywords:** climate reconstruction, dendroclimatology, pointer years, stable oxygen isotopes, volcanic eruptions

## Abstract

This study presents a new stable oxygen isotope chronology, covering the years 800–2000 AD, constructed using modern and subfossil wood derived from trees growing around Lake Schwarzensee in Austria. The climatic signal imparted in the chronology is conditioned mainly by the direct influence of environmental factors on the isotopic signature of source water, which in turn is regulated by evaporation and condensation mechanisms. The second driver of stable oxygen isotope is the physiological response of trees to changing weather conditions, most importantly rates of transpiration. The chronology of stable oxygen isotopes corresponds well with both temperature (r = 0.485; p < 0.05) and total precipitation (r = −0.548; p < 0.05) during the growing season (May–September). This mixed signal results from the fact that the relationship between the content of stable oxygen isotopes and the influence of climate is multifactorial. Moreover, the effect exerted by meteorological conditions on stable isotope ratio changes over time. This is most probably linked to interannual variation in climatic and environmental factors.

## Introduction

Stable isotope studies are an effective method that could be applied for palaeoclimatic reconstructions. However, this approach requires the use of appropriate research material. Wood is an excellent archive of environmental changes because climate conditions affect physiological mechanisms of plants and in this way influence isotopic composition of wood (Fritts 1976; Roden et al. 2000; Gagen et al. 2011). Due to the fact that wood has annual growth rings formed in consecutive years, it is possible to monitor climate with an annual resolution (Speer 2010). For this purpose, dendrochronological scales are used. They can connect the data over several thousands of years depending on chronology involved (Hughes 2011). However, the wood utilized during such studies must have a sufficiently strong climatic signal, and therefore it must come from sites characterized by a significant and undisturbed impact of meteorological factors on biological processes of trees (Briffa and Jones 1990).

The spruce chronology from the area of the Alpine Schwarzensee Lake used in this study tracks various climate data (Kłusek et al. 2015, 2019). This chronology comes from a mountainous region that is free from direct anthropogenic pressure. Therefore, the growth of trees in this habitat should be associated only with local edaphic and climatic factors. Nevertheless, the Schwarzensee region is also influenced by volcanic events taking place far from the lake but affecting weather of this area (Kłusek et al. 2015). Characteristic phenomena connected with volcanic eruptions significantly decreased temperature and solar radiation, as well as increased air humidity and precipitation. On the other hand, the area of wood origin has a very small geographic range, so all trees were subjected to the same meteorological conditions during their life and, owing to this, the climatic signal of the Schwarzensee chronology ought to be highly homogeneous. In addition, the construction of the chronology used only Norway spruce wood, making the reactions of the trees to climatic and environmental drivers species-specific.

The usefulness of this Schwarzensee chronology for isotopic studies is helped by the shallowrooting of Norway spruce (Schweingruber 1993). As a result, the trees have limited access to groundwater. This is beneficial because groundwater typically contains mixed precipitation from various years and months (McCarroll and Loader 2004). Additionally, the penetration of rainwater is limited in Schwarzensee area because much of it runs off the steep slopes and does not infiltrate deep into the soil. Furthermore, the Schwarzensee region is characterized by humid conditions with precipitation maxima occurring during the growing season. Increased precipitation and air humidity may also be related to the impact of volcanic activity, which simultaneously contributes to a decrease in temperature and solar radiation (Battipaglia et al. 2007) in the area of Schwarzensee Lake (Kłusek et al. 2015). In view of these factors, trees mainly obtain water that falls at the time of rainfall occurrence. This is a very favourable aspect for stable oxygen isotopic analysis.

The Schwarzensee chronology meets all the requirements to accurately reflect the palaeoclimate of the area. Therefore, the chronology has been subjected to various types of physico-chemical analyses in order to obtain a long-term data set. This is a very desirable approach since reconstructions based on multiple proxies ensure more precise results (McCarroll et al. 2003). So far, the width of annual growth rings (Grabner et al. 2006), the maximum density of latewood (Kłusek et al. 2015; Kłusek and Grabner 2016), and the content of stable carbon isotopes (Kłusek and Pawełczyk 2014; Kłusek et al. 2019) in growth-rings have been measured. All analyses to date have focused on determining the relationships existing between individual wood parameters (growth ring width, maximum density of latewood, stable carbon isotope ratio) and meteorological factors (temperature, precipitation, solar radiation). The results obtained so far will be used to identify past environmental changes in the area extending around Schwarzensee Lake. This paper is a further step towards reconstruction of the thermal conditions of the Schwarzensee Lake region. The article presents measurements and interpretations of stable oxygen isotopes. The aim of the study was to establish a new stable isotope chronology and then to define the influence of climatic drivers on the oxygen isotope content of wood.

## Theoretical Background

The relationship between climate and stable isotope signature of wood is well-defined by an existing model of oxygen isotopic fractionation (Gagen et al. 2011). This model indicates that the oxygen used by plants originates from water, and the sources of water for trees are soil moisture and precipitation. Xylem water, which moves up the stem and into the leaves, has the same isotope ratios as soil water. However, the isotopic composition of water changes due to discrimination processes that occur both before and after its incorporation into the plant tissue (Barbour et al. 2001).

The enrichment or depletion of the source water in heavier stable isotopes occurs first as a result of evaporation and condensation taking place in the vicinity of the tree. An important role in the isotope fractionation during this hydrological cycle is played primarily by temperature and, to a lesser extent, the relative humidity of the air, which is directly linked to precipitation. When the temperature is high and the humidity is low, evaporation rises. Because lighter isotopes evaporate faster, the source water is therefore relatively enriched in heavier isotopes. As an effect, the water absorbed by plants contains more ^18^O. At the same time, the evaporation leads to the light isotope content of water vapor being more abundant. Hence, enhanced condensation associated with the decrease in temperature and increase in humidity, reduces the ^18^O concentration of soil water and consequently contributes to the depletion of ^18^O in wood (Gessler et al. 2014).

In turn, the main physiological mechanism of isotope discrimination in plants is connected with stomatal activity, which in turn is primarily determined by water availability. Nevertheless, temperature fluctuations also exert some effect on opening and closing of stomatal apertures. Low temperature combined with high humidity and precipitation stimulate stomatal opening because under these conditions the water loss by the plant is substantially limited due to a small water vapor pressure difference between the leaf intercellular spaces and the ambient air. Since lighter isotopes are favoured in the transpiration process, the intensification of stomatal conductance causes the water in the leaf to be enriched in ^18^O (McCarroll and Loader 2004; McCarroll and Loader 2006).

However, the changes in the proportion of stable isotopes appearing as a derivative of stomatal opening or closure are always counteracted by the convection of isotopically lighter soil water through the transpiration flow (Barbour 2007). As a consequence of mixing of water from these two different sources, during the backward diffusion from the sites of transpiration to the rest of the leaf, the content of the ^18^O isotope is reduced. This reaction, known as the Péclet effect, dampens the signal imparted in the leaf (Gagen et al. 2011). Therefore, sucrose synthesized during assimilation is depleted in ^18^O in comparison with water from transpiration sites. Exchange with xylem water progressing during cellulose and lignin formation within the stem leads to a further lowering of the content of heavier isotopes (Gessler et al. 2014).

## Material and Methods

### Wood Origin and Site Condition

A chronology constructed on the basis of subfossil and modern wood from the area around Lake Schwarzensee (Grabner et al. 2006) was used for isotopic analysis in this study. Schwarzensee is a small mountain lake located in the Dachstein Mountains region of Austria (47°31 ′N, 13°49 ′E, 1450 m.a.s.l.). Subfossil wood composing this chronology was excavated from the bottom of the lake, while modern samples were taken from trees growing on the surrounding mountain slopes. Contemporary samples were used to extend the chronology to the present day, as the wood deposited in the lake did not cover this period. Furthermore, samples from living trees allowed climate data to be linked to isotopic record of wood.

The Schwarzensee area is characterized by long, cold winters, and short, cool to mild summers. Only three months of the year (June, July, August) have an average temperature above 10°C, and the five coldest months (November, December, January, February, March) have average temperatures below 0°C. The Schwarzensee vicinity distinguishes itself by high precipitation levels due to orographic lift. This region receives an average rain-equivalent of 1720 mm of precipitation per year. Rainfall occurs mostly in the warmer months (June, July, and August) (Auer et al. 2007; Efthymiadis et al. 2006).

### Sample Preparation

Norway spruce wood (*Picea abies* (L.) Karst.) of five contemporary and 39 subfossil samples was chosen for isotopic research. For analyses only samples characterized by a good preservation state were selected. Visible fragments of reaction or juvenile wood were also omitted. The width of growth-ring was measured using the LINTAB device and TSAP Win computer program (Rinn 2003). The assignment of tree-rings to the proper calendar year was done on the basis of the comparison with the existing master chronology (Grabner et al. 2006). Measurements of stable oxygen isotopes were carried out within consecutive rings. For this purpose, the samples were split into individual rings and then cut into thin slivers with a scalpel. The rings were not divided into early- and latewood zones but were utilized in their entirety for the research. Measurement of the whole growth-ring may be biased by the reserve materials which can contribute in the formation of earlywood. However, in the case of conifers originating from high-mountain areas the participation of the stored substances is very low and has a negligible influence on the results obtained (Belmecheri et al. 2022). Therefore, taking into account the fact that Lake Schwarzensee is located almost at an elevation of the regional timberline, it was decided to apply the whole growth-ring for the stable oxygen isotope research. Separated rings were pooled to eliminate individual variability observed between the particular trees and to ensure the appropriate amount of the wood. Averaged samples were composed of equally aged wood and were combined from four various trees weighted in equal proportions. This number of trees provides a representative Expressed Population Signal – EPS > 0.85 – regardless of the ecological and climatic conditions of environment in which plants grew (Dorado Liñán et al. 2011; Belmecheri et al. 2022).

The procedure of α-cellulose extraction was conducted in accordance with the modified Green (1963) method. For the removal of lignin the samples were treated in water solution of sodium chlorite and acetic acid (in proportions of 2.5 g NaClO_2_, 1.7 mL 80% CH_3_COOH and 175 mL H_2_O for 1 g of the sample). This preparation was carried out in an ultrasonic bath at 70°C and was repeated seven times at hourly intervals. After rinsing the samples with boiling deionized water until reaching neutral pH, the remains of lignin and hemicelluloses were eliminated from the wood by sodium hydroxide solutions. First 10% NaOH was added for the samples placed for 45 minutes in an ultrasonic bath with the temperature of 70°C (75 mL of 10% NaOH for 1g of the sample). Afterwards, the samples were flushed with boiling deionized water and later, they were subjected to 45-minute reaction with 17% NaOH in the ultrasonic bath of room temperature (67 mL of 17% NaOH for 1 g of the sample). Subsequently, the samples were ground in a mortar and finally, they were dried at the temperature of 70°C on a hot plate.

### Stable Isotopes Measurement and Chronology Construction

The content of stable isotopes was measured within three subsamples of wood originating from the same year. Alpha-cellulose was packed into silver foil capsules and subjected to “on-line” pyrolysis at 1300°C. IAEA Spruce, Rsta, and laboratory standard GdCell were used as reference materials to calibrate the sample isotopic composition versus the international Vienna Standard Mean Ocean Water (VSMOW). An EuroVector elemental analyser was directly connected with a continuous flow isotope ratio mass spectrometer IsoPrime EA-CF-IRMS. The precision of this method is 0.3‰ for oxygen stable isotope analyses. Measurement results were averaged within individual years and then directly employed to construct a new isotope chronology.

### Correlation with Climate and Volcanic Eruptions

The chronology of stable oxygen isotopes was compared with climatic data. To accomplish this, correlation coefficients (r) and response function values were calculated using DENDROCLIM 2002 software (Biondi and Waikul 2004). These calculations were carried out for individual months from the previous August to October of the current year. This period was chosen to take into account the influence of environmental conditions at the end of the growing season in the year preceding the formation of the current growth-ring. The meteorological data were taken from the HISTALP database (https://www.zamg.ac.at/histalp/index.php). The maximum available length of the meteorological record was employed for individual weather parameters: for temperature during 1780−2000 AD, for the sum of precipitation from 1800−2000 AD, and for solar radiation during 1884−2000 AD. In the case of temperature and precipitation time series, measurements were averaged for individual months and gridded for the area of Schwarzensee Lake by the application of data from the 1/6th of a degree grid box centred at 47°35′N, 13°45′E (Efthymiadis et al. 2006; Chimani et al. 2013). Values of solar radiation were from the meteorological station at Kremsmünster (48°06′N, 14°13′E, 389 m.a.s.l.), situated 96 km from the study area and at a much lower altitude (Auer et al. 2007).

To determine the influence of individual climatic factors on the content of stable oxygen isotopes in wood, additional analyses were carried out. For this purpose, the years with the highest and lowest values of δ^18^O were identified as pointer years. To detect these years, 5-year running mean (RM) and standard deviation (SD) were calculated. The arbitrarily established critical level of standard deviation (CL) appearing in the equations was assigned a value of 1. Negative pointer years were designated if they were less than: (RM − (CL × SD)) and positive pointer years were those greater than (RM + (CL × SD)) (Treydte et al. 2001). Next, meteorological measurements of temperature, precipitation, and solar radiation were separated into groups corresponding to positive, negative and remaining years. Finally, the Student t-test was applied to define whether significantly different climatic parameters existed between the pointer years and remaining years.

Calculated pointer years were also used to analyse the impact of large volcanic eruptions on the content of stable oxygen isotopes in wood. Severe volcanic events periodically alter weather conditions, even on a global scale. Therefore, pointer years were matched with strong volcanic eruptions characterized by an explosivity index equal to or greater than 4 (Siebert and Simkin 2002). Data available on the website https://volcano.si.edu/ were applied for the analysis. The comparison was performed for volcanoes located both in nearby and distant territories, with lags of up to 2 years between eruption and resulting pointer years considered.

## Results

**Figure 1:**
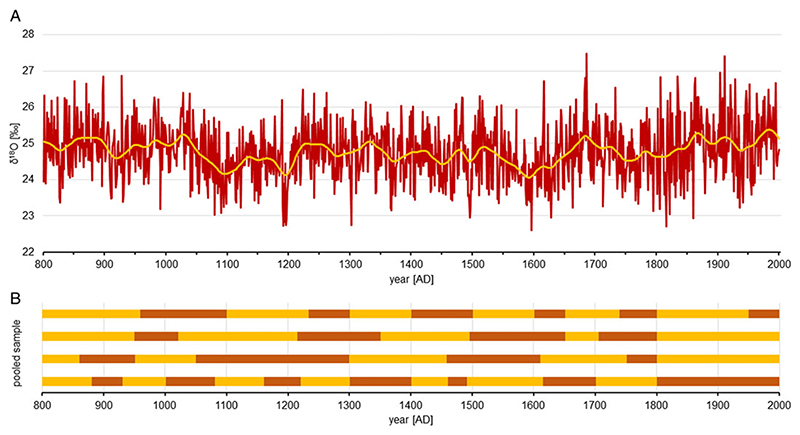
A. Schwarzensee stable oxygen isotope chronology and the chronology smoothed using spline fit with 50% variance cutoff at a wavelength of 50 years. B. The time spans of samples. Each of the four horizontal bars is divided into segments corresponding to individual trees. Colors indicate particular trees—a tree is darker or lighter than the previous one. (Please see online version for color figures.)

This study provides a new stable oxygen isotope chronology encompassing the years AD 800−2000. Over this time, there were clear long-term trends observed in the isotope content of the analyzed wood. Decreased δ^18^O was observed particularly in the intervals AD: 1050−1220 and 1550−1650, while increased values were present especially in the periods AD: 850−900, 1000−1050, 1210−1260, 1300−1350, 1650−1700, and 1850−2000 (Figure 1).

**Figure 2:**
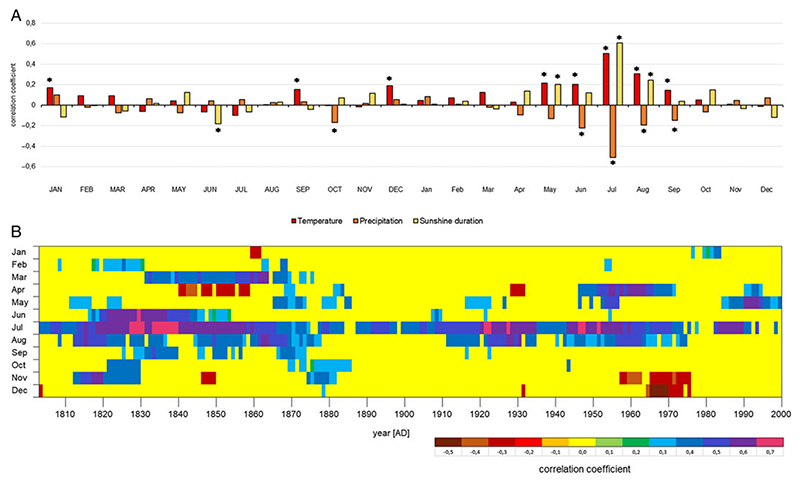
A. Bootstrap correlation coefficients calculated between mean monthly temperature, precipitation and solar radiation, and stable oxygen isotope chronology. Significant values at the 0.05 level are marked with an asterisk. Capital letters indicate months of the year preceding growth ring formation. B. Moving interval correlation coefficients computed between mean monthly temperature and stable oxygen isotope chronology. A base length of 25 years was progressively slid through the years from 1780 AD to 2000 AD. The figure shows significant values at the 0.05 level.

**Figure 3:**
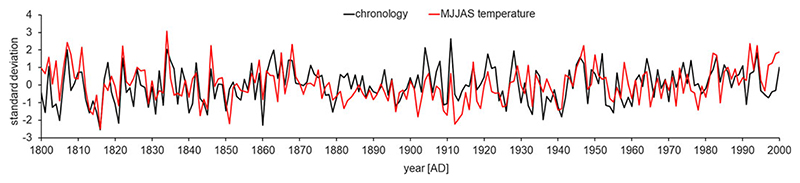
Comparison of normalized series of mean temperature averaged for May–September months (red line) with normalized Schwarzensee stable oxygen isotope chronology (black line).

The chronology was correlated with climatic data from the area of Schwarzensee Lake (Figure 2). Based on values of the correlation coefficient, statistically significant (p < 0.05) positive relationships existed between stable oxygen isotope ratio and May–September temperature, with r values of 0.214, 0.202, 0.502, 0.306, and 0.145 in respective months (Figure 3). For precipitation, statistically significant (p < 0.05) negative correlations occurred for June-September, with r values of −0.223, −0.511, −0.192, and −0.148, respectively. A positive correlation was also found between solar radiation and δ^18^O. Correlations between oxygen isotope ratio and solar radiation were statistically significant (p < 0.05) for May, July, and August, with r values of 0.203, 0.608, and 0.245, respectively.

**Figure 4:**
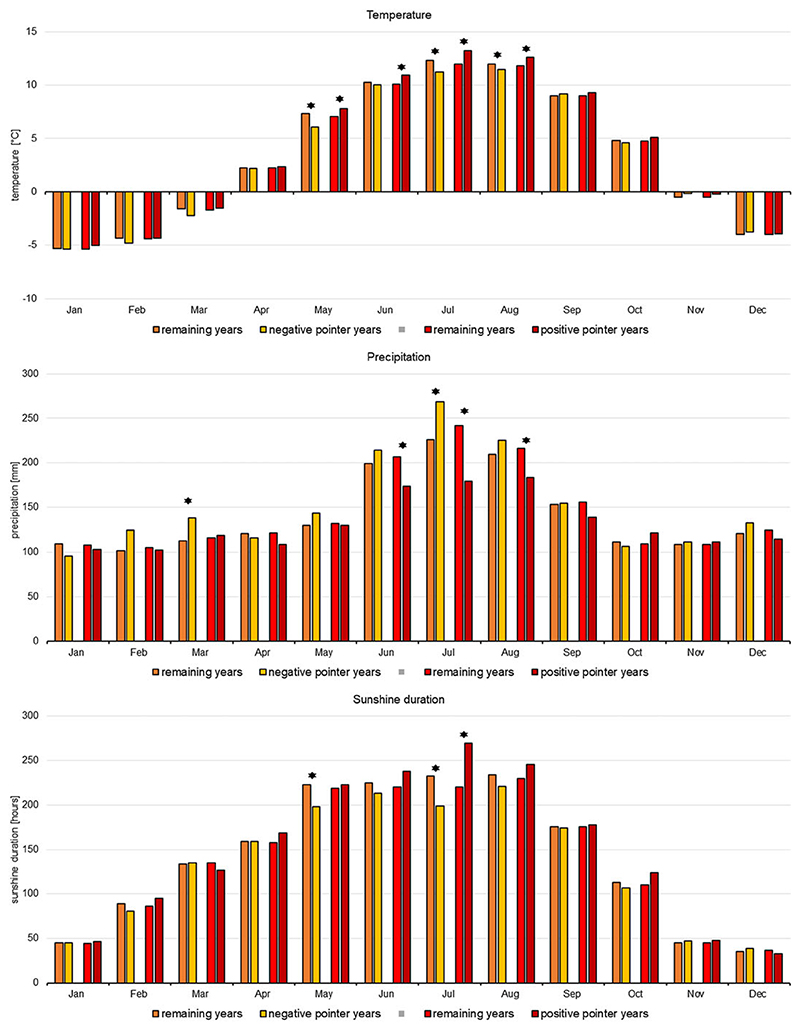
Mean monthly temperature, precipitation and solar radiation averaged for negative and positive pointer years and compared with these values averaged for remaining years. Student t-tests were applied to verify significant differences between groups of years. Differences significant at the 0.05 level are marked with an asterisk.

To confirm these observations and examine more closely relationships between weather and stable isotope ratio, an analysis of pointer years was performed. In total there were 204 positive pointer years and 183 negative pointer years (Table 1). The analysis of pointer years showed that significantly higher temperatures occurred for May, June, July, and August during positive pointer years, while significantly lower temperatures occurred for May, July and August during negative pointer years. Considerable links were also notified for the mean sum of precipitation, which was significantly higher in March and July in negative pointer years and lower in June, July, and August in positive pointer years. In contrast, solar radiation was higher in July during positive pointer years and lower in May and July during negative pointer years (Figure 4).

Pointer years were also used to evaluate whether weather changes associated with strong volcanic eruptions had a noticeable impact on the stable isotope content in the annual growth rings of trees growing in the region of Schwarzensee Lake. It was found that 62 negative pointer years and 73 positive pointer years coincided with volcanic explosions taking place either in the same year or in the 1−2 years preceding the pointer year (Table 1).

## Discussion

### Impact of Evaporation and Condensation Processes

Correlations with meteorological data obtained for the study area show that the elevated concentration of ^18^O in wood was related to increased temperature and decreased precipitation. These dependencies can be most accurately associated with the intensity of evaporation and condensation mechanisms. Lighter oxygen isotopes evaporate faster, while heavier particles remain in the source water, where they are taken up by the root system into plants (McCarroll and Loader 2006). Thus, the intensification of soil water evaporation, stimulated mainly by high temperature and to a lesser extent by low vapor pressure, led to a greater share of ^18^O in the examined wood. As suggested by the analysis of pointer years, the link between δ^18^O and temperature during the growing season was significant for negative as well as positive pointer years. Hence, both lower and higher temperatures by affecting evaporation rate, had a significant effect on stable oxygen isotope content in Schwarzensee wood.

The second phenomenon influencing the isotopic composition of source water, i.e., condensation, was characterized by similar relationships. Enhanced condensation reduces the ^18^O content of the growth rings because rainwater is enriched in ^16^O as a result of the preferential evaporation of the lighter ^16^O (Gagen et al. 2011). Therefore, lower temperature and higher moisture promoted condensation of water vapor and caused ^18^O depletion in the analyzed wood. Such a dependence is visible during pointer years significantly correlated with precipitation. In the case of negative pointer years, the decrease in δ^18^O was most probably induced by supplying plants with water from rainfall that was rich in ^16^O. In turn, during positive pointer years a small precipitation amount resulted in low condensation and high ^18^O concentration in the source water. As the analysis of the pointer years indicates, this second relationship was statistically significant for a larger number of months, so a precipitation decline had a more visible effect on oxygen stable isotope content than providing trees with greater water resources.

### Stomatal Activity, Péclet Effect, and Exchange with Xylem Water

Stomatal conductance, the next factor influencing composition of stable oxygen isotopes, is mainly conditioned by water availability. As a consequence, high rainfall and humidity allow intense transpiration. Because during transpiration, oxygen molecules that comprise the lighter isotopes are preferentially released, this process finally leads to the enrichment in heavier isotopes of water remaining in the leaf (Gagen et al. 2011). Therefore, enhanced precipitation causing an enlargement of transpiration rate should result in δ^18^O rise. However, obtained correlations between precipitation and the stable oxygen isotope content are negative for Schwarzensee chronology. Hence, it may be concluded that mechanisms governing isotope discrimination by stomatal opening and closure usually did not have a strong impact on the oxygen signature of Schwarzensee wood. As water resources are not a limiting factor for tree growth in the lake area, perhaps stomatal activity remained more or less constant and did not significantly contribute to changes in the oxygen stable isotope content of wood. The other explanation of this situation could be advection and mixing of source water which occurred in leaf and stem. These processes may have been so intense that leaf water enrichment was lost and this signal suppressed (cf. Treydte et al. 2014).

### Influence of Volcanic Events

On the other hand, it is likely that stomatal conductance was decisive for stable isotope composition in years when there was intense volcanic activity. In the course of this study, it was found that some volcanic events coincided with positive pointer years, which express themselves by increased ^18^O in the wood. During growing seasons distinguished by significantly lower temperature and solar radiation, as well as higher air humidity and rainfall, stomata could remain open to a greater extent. Stomatal conductance amplifies during these periods because the water loss is substantially limited due to a smaller water vapor pressure difference between the leaf intercellular spaces and the ambient air (Gessler et al. 2014). In turn, intensive transpiration increases the content of heavier isotopes in leaf water as mentioned above. In this way, the volcanic impact may contribute to the enrichment of wood in δ^18^O.

Other interactions between δ^18^O and volcanic activity may exist as well. Stomata open widely when air humidity is high, which could allow intense diffusion of ^18^O-depleted water vapor from ambient air into leaf tissue. Moreover, during years with volcanic eruption, the level of δ^18^O in water vapor may be additionally reduced as a result of lower temperature which generates higher humidity and rainfall (Battipaglia et al. 2007). These factors decrease δ^18^O values in leaf water, which is reflected in the organic matter formed at such times. The phenomena described explain the existing link between eruption events and negative pointer years.

However, it is also possible that decline in δ^18^O occurred in years when volcanic activity was weaker and had little effect on tree growth. In this case, interdependencies between weather and the ratio of stable isotopes could be controlled by the typical for this area, the previously described evaporation and condensation processes. In such a situation, the depletion of ^18^O in wood was caused by diminished evaporation and enlarged condensation which were stimulated in turn, by low temperature and high moisture level.

### Comparison with Stable Carbon Analysis

Similar conclusions confirming the variability in response of trees to climate and environmental factors can be drawn from previous studies. In former research, measurements of stable carbon isotope ratios were conducted using the same wood from Schwarzensee Lake (Kłusek et al. 2019). As in the case of oxygen, stable isotope studies of carbon allow the reconstruction of climatic conditions. Because organic carbon is derived from atmospheric carbon dioxide, the content of carbon isotopes in wood is therefore determined by the intensity of stomatal conductance and photosynthesis rate. When solar radiation and temperature are high but precipitation and humidity are low, assimilation is enhanced and the stomatal apparatuses are closed. This leads to rapid consumption of all the ^12^C available and preferred by the plant. As a consequence trees use more ^13^C, compared to cold and wet periods when photosynthesis level diminish but gas exchange through the stomata enhances enabling for greater carbon dioxide uptake. Performed research demonstrates that the stable isotope content of carbon in Schwarzensee wood is mainly dependent on temperature and solar radiation. These meteorological parameters have the strongest impact on the intensity of photosynthesis. In contrast, the activity of the stomata, which is regulated by water availability, is of lesser importance. Only during exceptionally dry seasons, namely during the hottest summer months, when trees are most susceptible to moisture deficit, was the reduced water supply to the plants the most likely reason for the closure of stomata and rising ^13^C content in wood. On the basis of performed studies it was concluded that the interdependencies between weather parameters and the carbon isotope ratio vary with time (Kłusek et al. 2019). The results obtained from isotopic analyses of carbon and oxygen are therefore consistent.

### Features of Schwarzensee Stable Oxygen Isotope Chronology

Obtained results prove that the chronology of stable oxygen isotopes corresponds well with both growing season temperature and precipitation. This mixed signal is due to the fact that the relationship between the content of stable oxygen isotopes in the annual growth rings and the influence of weather conditions is multifactorial. Moreover, the impact exerted by weather on stable isotope ratios changes over time. These fluctuations are most probably linked to variation observed in climatic and environmental factors that occurs from one year to the next. As a result of this research, it was concluded that oxygen isotopic composition of tree-ring cellulose usually represents a source water isotopic signature, modified to a greater or lesser extent by evaporative enrichment or by depletion proceeding during the condensation process. In contrast, isotope discrimination connected with stomatal opening and closure have a smaller effect on the proportion of stable oxygen isotope in the analyzed wood. The findings indicate also that the dependence on volcanic activity is strictly evident. Summing up, it could be stated that the most characteristic feature of the chronology is its unstable response to weather conditions through time and its changeable climatic signal. The relationship between weather and the ratio of stable isotopes was variable over time and dependent on the strength of individual meteorological factors.

## Conclusions

The climatic signal shown in an oxygen isotope chronology constructed using wood from the area of Schwarzensee Lake was conditioned mainly by the direct influence of environmental factors on the isotopic signature of source water, which in turn was regulated by evaporation and condensation mechanisms. The second main driver of oxygen isotopes was the physiological response of trees to changing weather, most strongly manifested by the varying intensity of transpiration. As a result, the Schwarzensee chronology recorded temporal fluctuations in isotope composition of the source water used by the plants, although the isotopic signature of soil water was to various degrees modified by trees in response to environment. Moreover, the most characteristic feature of the relationships observed between stable isotope content in wood and atmospheric phenomena is its variability over time. This instability probably results from the varying intensity of individual meteorological parameters in different years. This means that the new chronology provides a mixed signal, and therefore its application for palaeoclimatic reconstruction must be preceded by a thorough analysis of the factors governing stable oxygen isotope composition of wood.

## Supplementary Material

To view supplementary material for this article, please visit https://doi.org/10.1017/RDC.2022.91

Supplementary Material

## Figures and Tables

**Table 1:** Negative and positive pointer years calculated for the stable oxygen isotope chronology and compared with volcanic events. Normal font style denotes pointer years not related to volcanic events, italic corresponds to pointer years preceded by volcanic activity during the previous 1–2 years, and the bold values are pointer years coinciding in time with the volcanic eruptions (volcanic data are available on the website https://volcano.si.edu/).

Negative pointer years					Positive pointer years				
804	1107	1399	**1693**	*1987*	806	**1050**	**1262**	1552	**1846**
827	1114	1406	1698	**1991**	813	1053	1268	1559	1852
831	1117	1411	1703	1997	817	1058	1279	1567	**1857**
837	1122	1414	1710		820	1062	1284	*1571*	*1863*
847	1132	1420	*1713*		**823**	1070	1288	1584	1868
853	1135	1425	*1723*		830	1074	1293	1592	**1873**
859	1140	1428	1736		835	1078	1305	**1595**	**1893**
866	1144	1432	*1740*		**838**	*1081*	1319	1603	*1904*
879	1148	1438	*1745*		*842*	1084	1326	1607	*1908*
884	1151	*1443*	**1749**		845	1087	1333	1611	**1911**
896	1154	1446	*1756*		*851*	1092	1336	1616	**1917**
*901*	1165	*1452*	**1762**		*855*	1099	1344	*1623*	*1921*
923	1169	1456	**1766**		*861*	*1102*	1348	**1631**	1928
*936*	1175	1470	*1770*		864	*1106*	**1357**	1637	*1934*
940	1180	1475	1773		867	*1109*	*1361*	1644	*1939*
947	1185	*1478*	1776		**870**	1115	*1371*	1653	**1952**
**950**	1196	*1481*	**1779**		**874**	1120	**1380**	1659	**1956**
955	1206	1490	**1786**		880	1125	1384	1666	**1964**
959	**1210**	1496	**1795**		**885**	**1130**	1393	1669	1967
964	1213	*1511*	*1801*		*889*	1134	1397	1672	1971
967	1219	1515	*1816*		898	1146	**1400**	1676	**1976**
*971*	1242	1519	1821		905	1149	1403	1686	**1983**
991	**1245**	1523	**1825**		*916*	1155	1407	*1691*	**1994**
994	1249	1527	*1837*		*922*	*1159*	1410	*1696*	**2000**
997	1256	*1531*	1840		928	1167	1413	1702	
*1001*	1260	*1533*	**1845**		937	**1170**	1419	1706	
1004	1271	1543	1850		945	1173	1426	**1717**	
1007	1275	**1550**	**1854**		948	1177	**1430**	**1720**	
1017	1295	**1560**	**1860**		952	**1183**	1434	1726	
**1020**	*1302*	*1565*	1866		956	1189	1439	*1728*	
**1030**	1306	1569	*1879*		961	1194	**1444**	1733	
1036	1307	1579	**1888**		966	**1200**	1447	*1746*	
**1040**	**1310**	*1583*	1896		974	1208	1454	**1759**	
1043	1314	1596	*1906*		981	*1212*	1476	**1768**	
1051	1322	**1600**	1909		987	1217	1487	1774	
1054	1329	**1606**	**1926**		993	1223	1488	**1778**	
1060	1335	**1612**	**1933**		998	*1228*	1492	*1784*	
1063	*1342*	1624	1936		1006	1232	*1501*	*1788*	
1072	1346	1628	1941		1011	1238	1504	*1802*	
1079	1356	**1640**	*1948*		1018	1241	1512	1807	
1082	1359	1649	**1963**		1028	1244	1516	**1818**	
1085	1374	**1663**	**1966**		1031	1248	1525	**1822**	
1090	1383	**1667**	*1970*		1034	1252	*1536*	**1826**	
1094	1388	*1675*	1979		1039	1255	**1540**	1834	
1098	1392	1685	1984		1044	1259	1547	1842	
